# Chlorophyll a in lakes and streams of the United States (2005–2022)

**DOI:** 10.1038/s41597-024-03453-3

**Published:** 2024-06-12

**Authors:** Sarah A. Spaulding, Lindsay R. C. Platt, Jennifer C. Murphy, Alex Covert, Judson W. Harvey

**Affiliations:** 1 U.S. Geological Survey, INSTAAR, 4001 Discovery Drive, Boulder, CO 80309 USA; 2 U.S. Geological Survey, 1 Gifford Pinchot Drive, Madison, WI 53726 USA; 3 Consortium of Universities for the Advancement of Hydrologic Science, Inc., 1167 Massachusetts Ave. – Suites 418 & 419, Arlington, MA 02476 USA; 4 U.S. Geological Survey, 650 Peace Road, Dekalb, IL 60115 USA; 5 U.S. Geological Survey, 6460 Busch Blvd. - Ste. 100, Columbus, OH 43229 USA; 6 U.S. Geological Survey, 12201 Sunrise Valley Drive, Reston, VA 20192 USA

**Keywords:** Limnology, Hydrology

## Abstract

The concentration of chlorophyll *a* in phytoplankton and periphyton represents the amount of algal biomass. We compiled an 18-year record (2005–2022) of pigment data from water bodies across the United States (US) to support efforts to develop process-based, machine learning, and remote sensing models for prediction of harmful algal blooms (HABs). To our knowledge, this dataset of nearly 84,000 sites and over 1,374,000 pigment measurements is the largest compilation of harmonized discrete, laboratory-extracted chlorophyll data for the US. These data were compiled from the Water Quality Portal (WQP) and previously unpublished U.S. Geological Survey’s National Water Quality Laboratory (NWQL) data. Data were harmonized for reporting units, pigment type, duplicate values, collection depth, site name, negative values, and some extreme values. Across the country, data show great variation by state in sampling frequency, distribution, and methods. Uses for such data include the calibration of models, calibration of field sensors, examination of relationship to nutrients and other drivers, evaluation of temporal trends, and other applications addressing local to national scale concerns.

## Background & Summary

This data descriptor explains the recently published national harmonized dataset of chlorophyll *a*^1^ assimilated and curated from the Water Quality Portal (WQP)^2^ and United States Geological Survey (USGS) National Water Quality Laboratory (NWQL, Denver Federal Center, Building 95 Lakewood, CO). The dataset represents an 18-year record (2005–2022) of samples of phytoplankton (suspended algae) and periphyton (benthic algae) from lakes, streams, rivers, reservoirs, canals, and estuaries in the United States and its territories (continental US, Hawaii, Alaska, American Samoa, Guam, Northern Mariana Islands, Puerto Rico, and the U.S. Virgin Islands). Chlorophyll *a* is one of many pigments found in photosynthetic organisms, functioning to 1) absorb light energy, 2) transfer energy to the chlorophyll reaction center, and 3) separate charges within the reaction center to promote biosynthesis^3^. There are many types of chlorophyll molecules, including chlorophyll *a, b, c*^1^*, c*^2^, *d*, and others, each with differing molecular sidechains that absorb light at specific wavelengths. While different algal lineages contain different chlorophylls and accessory pigments, chlorophyll *a* is found in all algae, including Cyanobacteria (blue-green algae), Chlorophyta (green algae), Cryptophyta (cryptophytes), Chrysophyta (yellow-brown algae), Bacillariophyta (diatoms), Dinophyta (dinoflagellates), and Phaeophyceae (brown algae)^4^.

The concentration of chlorophyll *a* is reflective of ecosystem processes and metabolism, including primary production and respiration^5,6^, which together can be used to estimate the rate of change in organic carbon or, more specifically, to help estimate the rate of change of biomass of the autotrophs^7,8^. Chlorophyll concentration is also a primary component, along with nitrogen and phosphorus concentrations, in classifying the trophic status of waters^9–12^. Yet, the concentration of chlorophyll *a* is only a rough proxy for algal biomass because cell chlorophyll content is variable across species, nutrient concentration, light regime, temperature, and cell condition^13–17^. Nevertheless, chlorophyll *a* concentration is widely used as a surrogate of algal biomass because of the difficulty of measuring algal biomass directly.

Different primary habitats support different types of algae and separate units of measure are needed to report concentration between habitats. Lakes and large rivers typically support phytoplankton, which are algae suspended in the water column. Phytoplankton are measured in units based on volume (µg/L). Headwater, shallow (wadeable), and clear streams and rivers support periphyton attached to the benthos, where the benthos is illuminated^18^. Periphyton are measured in units based on area (mg/m^2^). Our dataset includes measures of chlorophyll from both phytoplankton and periphyton, yet these data are not directly comparable. Phytoplankton samples consist of a grab sample of a volume of water^18^. The sample volume collected, and later filtered, is adjusted for the concentration of phytoplankton. For example, large volumes (~1–5 L) are required to obtain an adequate number of algal cells from oligotrophic lakes, while algal blooms that are dense with cells might have more than enough algal cells in 20 mL. In contrast, periphyton samples are collected by removing (scraping) a known surface area of the benthos. Concentrations of periphyton can reach very high levels, however, and a high benthic concentration of chlorophyll does not necessarily reflect high growth rates, because growth rates vary as cells accumulate and senesce over time. High flow causes mobilization of the riverbed that can scour and remove the accumulated periphyton.

Analysis of chlorophyll *a* is inexpensive, with minimal processing required^19^. Thus, its measurement has become a routine component of water quality programs at local, state, tribal, and federal resulting in an extensive, although unchecked, dataset. We report on the harmonized dataset was for calibration of process models and machine learning models to understand harmful algal bloom (HAB) events, particularly in rivers. River HABs have been historically understudied, although they are gaining more attention as the occurrence and distribution of algal toxins and toxigenic taxa are being realized^20^. We also have the goal of providing the largest source of harmonized data for calibration of remote sensing and other model types for U.S. water bodies^21^. Yet, there are a multitude of other uses of the data, such as calibrating field sensor data^22^, examining the relationship to nutrients, evaluating temporal trends^23^, and other applications to address local to national scale questions.

Many of the available datasets that include chlorophyll *a* are focused on lakes^24^. Others, including the AquaSat pipeline and dataset^25^, were specifically developed to train and validate remote sensing models by providing matched measures of total suspended sediment, dissolved organic carbon, chlorophyll *a*, and Secchi depth with remotely sensed spectral reflectance. Notably one of largest and most comprehensive datasets of chlorophyll *a*, LAGOS-NE, combines land use, geologic, climatic, and ecological context for lakes in 17 states^26^. Other datasets include international records, such as a literature survey of global lakes combining data for chlorophyll *a*, associated water chemistry, and lake morphometry for nearly 12,000 lakes^27,28^ and a coordinated field campaign by scientists in 27 countries that sampled 369 European lakes for chlorophyll and other parameters^29^. The coordinated campaign used a standardized approach to sampling and analysis of physical, chemical, and biological parameters that resulted in a unique dataset, including multiple algal pigments and cyanotoxin concentrations at each site. A few notable exceptions to lake-dominated datasets are the long-term record of chlorophyll *a* concentration in the Mississippi River near its terminus^30^ where the data are high-frequency and long-term (1997–2018), although from a single station. Additionally, the Long-Term Resource Monitoring (LTRM) program, which is a joint monitoring effort by the USGS and US Army Corps of Engineers (USACE), samples multiple reaches and pools of the upper Mississippi River for ecological endpoints, including chlorophyll.

While our dataset includes chlorophyll *a* from phytoplankton in lakes and estuaries, the largest number of records is from rivers. Within river collections, chlorophyll *a* data may be from phytoplankton or from periphyton, or both. Periphyton that is disturbed by high flows and entrained into the water column mixes with phytoplankton that are present. Determining algal source can be difficult, but approaches include species identification, taxon guild, population time series, and aggregation of sensor data^31,32^. Nevertheless, rivers tend to be dominated by either phytoplanktonic or periphytic growth at any given time, with deeper, turbid rivers favouring phytoplankton and shallow, clear rivers favouring periphyton^31^.

In general, samples of water (phytoplankton) or benthic surfaces (periphyton) are collected in a quantitative manner^18^. Samples are filtered in dark conditions and transported on dry ice to a laboratory for analysis. Again, in dark conditions, chlorophyll *a* is extracted from the filter using a solvent, usually acetone or ethanol. The laboratory protocols used to analyze chlorophyll a are spectrophotometry, fluorometry, and high-performance liquid chromatography (HPLC)^19,33–38^. HPLC is considered the most accurate method, but it is cost-prohibitive for widespread application. Spectrophometry is well-suited to moderate levels of chlorophyll *a*, while fluorometry is more sensitive to low concentrations^37^. Both methods are better suited to routine measurements than HPLC. A limitation with all methods, however, is that different algal classes contain differing amounts of the various chlorophylls, accessory pigments, and degradation molecules^31^, and knowledge of algal taxonomic composition is helpful in interpreting changes in chlorophyll concentration.

Environmental Protection Agency (EPA) methods 445 and 446^19,20^ are the standard approaches for determining chlorophyll concentrations and require two separate measurements (instrument readings) to compute concentrations of three pigments: uncorrected chlorophyll *a*, corrected chlorophyll *a*, and pheophytin. First, “uncorrected chlorophyll *a”* is based on the initial optical measurement of the sample (EPA 445, Section 12.1). The pigment chlorophyll *a* and its degradation pigment, pheophytin, are included in this measure. A second optical measurement is made after acidification of the sample, as acidification results in the loss of the central Mg^+^ atom, converting chlorophyll to pheophytin. “Corrected chlorophyll *a*” (EPA 445 Section 12.2) and “pheophytin” (EPA 445 Section 12.3) are based on calculations to estimate the amount of each pigment in the original sample using both optical measurements. Note that the calculations depend on instrument sensitivity settings and measurement of the ratio of initial chlorophyll *a* reading to acidified reading. Importantly, simply subtracting pheophytin concentration from uncorrected chlorophyll *a* concentration *is not equivalent to* corrected chlorophyll *a*^19^. This national harmonized dataset distinguishes between the three pigment types obtained from these standard methods and their importance.

EPA method 445 includes discussion of a modified method, using a narrow band pass filter. The narrow excitation and emission filters were evaluated for their ability to eliminate spectral interference (caused by chlorophyll *b* and pheophytin) and developed into the modified method described in EPA method 445. The technique was determined to be an alternative to the standard fluorometric method^19^. However, values obtained from the EPA 445 method for corrected chlorophyll *a* based on acidification and corrected chlorophyll *a* by the modified method are coded in the same way (they have the same USGS parameter codes and same WQP Characteristic Names), yet these values may not be comparable in natural waters.

Finally, many datasets do not distinguish or report the different pigment values (chlorophyll *a*, uncorrected for pheophytin; chlorophyll *a*, corrected for pheophytin; pheophytin) that are obtained from standard methods^15,19,34–37^. Due to their importance and influence on analysis and interpretation of the data, this effort distinguishes pigment types obtained from standard methods.

## Methods

**Fig. 1:**
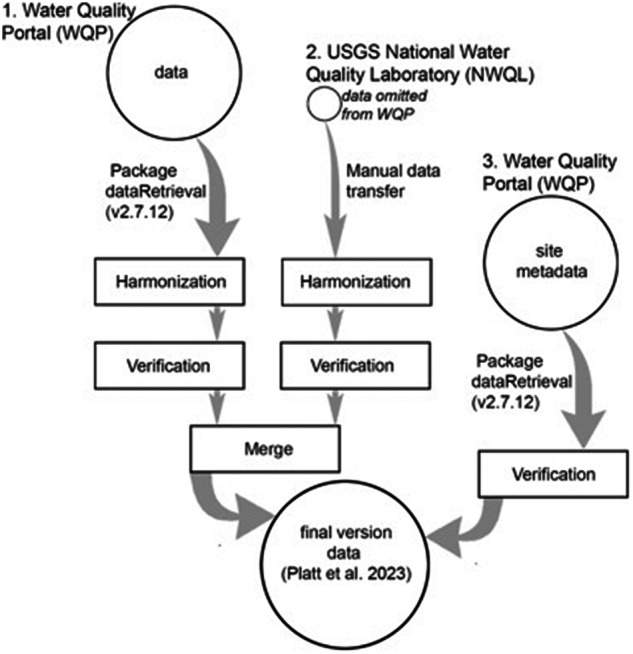
Workflow diagram indicating the sources of data (Water Quality Portal data, USGS National Water Quality Laboratory, and Water Quality Portal metadata), harmonization and verification steps, and final product. The WQP integrates publicly available water quality data from the USGS National Water Information System (NWIS), the EPA Water Quality Exchange (WQX) Data Warehouse, and the USDA ARS Sustaining The Earth’s Watersheds - Agricultural Research Database System (STEWARDS) https://www.waterqualitydata.us/.

Data were obtained via a custom data pipeline, which included computer code for executing a series of processes to obtain and manipulate data from one or several sources^1,39^. Data were obtained from the WQP^2^ and USGS NWQL. Figure 1 gives an overview of the data retrieval, harmonization, and verification workflow. The WQP database aggregates records from many sources, including the Water Quality Exchange (WQX) (www.epa.gov/waterdata/water-quality-data-upload-wqx), an EPA website that houses EPA, other federal agencies, universities, citizen, and other data sources. WQP also includes data from the USGS, National Water Information System (NWIS) (waterdata.usgs.gov/nwis). We discovered that some records from NWQL had not been submitted to NWIS, so we added those NWQL data to the larger dataset. The R programing language, RStudio, and the R package ‘targets’ were used to develop and run the data pipeline^40–42^.

### Retrieval

Data retrieval methods were different for the WQP and the NWQL records. The USGS R package, dataRetrieval (v2.7.12)^42^ was used to retrieve records from the WQP. The WQP uses “Characteristic Names” to distinguish between parameters. We identified ten chlorophyll-related parameters to use for retrieval (Table 1). First, site types (lake, stream, estuary, facility) with at least one sample for one of the ten chlorophyll parameters were identified. Then data were downloaded, and chlorophyll parameters were mapped to three pigment types (Table 1). Chlorophyll records from NWQL were requested from laboratory staff and received by the project team as comma separated values (CSV) files. NWQL provided pigment type in the parameter name. However, only two pigment types were available from NWQL, “chlorophyll a, corrected for pheophytin” and “pheophytin”.

**Table 1:** Ten parameters (Characteristic Names) selected for retrieval from WQP.

WQP Characteristic Name	Pigment
Chlorophyll*	Chlorophyll *a*, corrected for pheophytin
Chlorophyll *a*	Chlorophyll *a*, corrected for pheophytin
Chlorophyll *a* - Periphyton (attached)	Chlorophyll *a*, corrected for pheophytin
Chlorophyll *a* - Phytoplankton (suspended)	Chlorophyll *a*, corrected for pheophytin
Chlorophyll *a*, corrected for pheophytin	Chlorophyll *a*, corrected for pheophytin
Chlorophyll *a*, free of pheophytin	Chlorophyll *a*, corrected for pheophytin
Chlorophyll *a*, uncorrected for pheophytin	Chlorophyll *a*, uncorrected for pheophytin
Pheophytin	Pheophytin
Pheophytin *a*	Pheophytin
Phaeophytin - Periphyton (attached)	Pheophytin

The ten Characteristic Names are mapped to three pigment types 1) chlorophyll *a*, corrected for pheophytin, 2) chlorophyll *a*, uncorrected for pheophytin, and 3) pheophytin. Note the alternate spelling of pheophytin, “phaeophytin”.

*Some samples (from ‘org_cd ‘of “MDE_FIELDSERVICES_WQ” and “21FLHILL_WQX”) reported ‘Chlorophyll’ values that were a sum of chlorophyll *a* and chlorophyll *c*.

### Harmonization

WQP and NWQL data were mapped into common columns, units, and categories, without removal of records (Tables 2, 3). WQP retrieval contain many columns of metadata. For this effort, we focused on the columns containing the most pertinent information for using these measurements for model calibration and data analysis.

**Table 2:** WQP data were mapped to columns in the harmonized dataset.

WQP Column Names	Dataset column name	Values
Source	source	“WQP” or “NWQL”
MonitoringLocationIdentifier	site_no	Unique identifier
ActivityStartDate	date	YYYY-MM-DD
ActivityStartDateTime	date_time	YYYY-MM-DD HH:MM:SS in UTC
ResultMeasureValue	result_va	pigment concentration
ResultMeasure.MeasureUnitCode	result_units	µg/L, mg/m^2^
ActivityDepthHeightMeasure.MeasureValue	sample_depth	meters
	censored_cd	*
ResultStatusIdentifier	remark_cd	Indicates the acceptability of result, with respect to QA/QC
ResultAnalyticalMethod.MethodName	method_cd	**
ResultCommentText	result_qualifier	Any issue that affects result
HydrologicEvent	hydro_event_cd	i.e. algal bloom, storm

*The censored code (censored_cd) was derived from result_va and result_cd fields. If result_cd was equal to ‘Present Below Quantification Limit’, ‘*Present < QL’, or ‘Below Reporting Limit’, then a value of ‘ < , no value’ was entered for censored_cd. If result_va was equal to ‘*Non-detect’, ‘*Not Reported’, ‘ND’, or ‘##(Censored)’, then a value of ‘Censored, unknown’ was entered for censored_cd. Other potential values include the symbols ‘<’ or ‘>’, which were removed from result_va and moved to censored_cd.

**The field for method included a long list of fluorometric and spectrophotometric methods based on EPA 445. It also included reference to specific projects (i.e. “CONTACT VADEQ FOR DETAILS”, “LAKEWATCH-CHL”, and others) and “NA”.

**Table 3:** NWQL data were mapped to columns within the harmonized dataset in a similar manner to WQP data (Table 2).

NWQL column names	Mapped to column name	Values
Source	source	NWQL
Site	site_no	USGS-STATIONID
Date	date	YYYY-MM-DD
Time	date_time	YYYY-MM-DD HH:MM:SS
FINAL	result_va	pigment concentration
UNITS	result_units	µg/L, mg/m2
	censored_cd	*
LABCODE	method_cd	Following EPA 445
QUALIFIER	result_qualifier	Any issue that affects result

NWQL used the field ‘parameter name’ for the two pigment types measured: “chlorophyll a, corrected for pheophytin” and “pheophytin”.

*Censored_cd was derived from the NWQL “FINAL” column. Information from “FINAL” was parsed into three columns: result_va, censored_cd, and remark_cd. Symbols for ‘<’ or ‘>’ that appeared in ‘FINAL’ were extracted and placed in censored_cd. Any additional non-numeric value was extracted from ‘FINAL’ and placed in remark_cd (only values of “E” for estimated values appeared in this dataset).

The WQP records include fields with specific values for source of the data (source), site number (site_no), date (date, reported in UTC) date and time (date_time, reported in UTC), value of the result (result_va, concentration of pigment), units of the result (result_units, as µg/L or mg/m^2^), depth of sample collection (sample_depth, meters), censor code (censored_cd), remarks (remark_cd, indicate QA/QC status), method reported by the laboratory (method_cd, values that indicate spectrometry of fluorometry following EPA Method 445), comments concerning results (result_qualifier, comments concerning issues that may affect pigment concentration), and hydrologic events (hydrologic_event_cd).

Several fields contained values that were nonsensical or units that required harmonization. The calendar date on which the sample was collected is reported. The local time that the sample was collected was converted to UTC in the POSIX standard format, ‘YYYY-MM-DD HH:MM:SS’. Records with analytical methods that could not be tied to chlorophyll (Organophosphorus Compounds by Gas Chromatography: Capillary Column Technique, Nitrite Nitrogen by Spectophotometry, Conductance, Nutrient analyses, Phosphorus by Colorimetry, Turbidity by Nephelometry, 5 Day Biochemical Oxygen Demand, Alkalinity in Water by Titration, Nitrate-Nitrite Nitrogen by Colorimetry, 2540 D ~ Total Suspended Solids in Water, Total Kjeldahl Nitrogen by Colorimetry, Biochemical Oxygen Demand (BOD(5)), 10-115-01-1 F ~ Total Phosphorus, Manual persulfate digest and 2320 B ~ Alkalinity by Gran Titration) were omitted.

The ‘censored_cd’ column was created by identifying and grouping values from the ‘result_va’ and ‘result_cd’ columns into different censored value types: if ‘result_cd‘ was one of ‘Present Below Quantification Limit’, ‘*Present <QL’, or ‘Below Reporting Limit’, then a value of ‘<, no value’ was included for ‘censored_cd’. Any record with a ‘result_va’ matching one of ‘*Non-detect’, ‘*Not Reported’, ‘ND’, or ‘##(Censored)’ was given a ‘censored_cd’ value of ‘Censored, unknown’. The remaining values contained the symbols ‘<’ or ‘>’, which were unpaired from the result value itself and moved to the ‘censored_cd’ column.

Units for phytoplanktonic pigment concentration were variable (mg/l, ppm, ug/l, mg/m3, ppb, mg/cm3, ug/ml, mg/ml) and were converted to µg/L. Similarly, units for periphyton pigment concentration (g/m2, mg/cm2, mg/m2, ng/cm2, ug/cm2) were converted to mg/m^2^. Records with inappropriate units (NA, %, IVFU, mg, None, NTU, RFU, umol/m2/s, volts) were omitted.

Depth of sample collection ranged up to 376 m, but any record with a reported depth over 10 m was omitted. While the deep chlorophyll maximum is a well-known feature of lakes^34^, records from such depths are potentially misleading for modelling and remote sensing efforts. Sample depths with “NA” were retained with the assumption that most sample depths would be near the water surface. We noted that a large percentage (~40%) of chlorophyll data in NWIS were characterized as “preliminary”, meaning they had not been checked by a data steward. These records were all retained.

The NWQL retrieval contained a limited set of columns provided by the laboratory. Columns included: source of the data (‘source’, NWQL), site number (‘site_no’, USGS STATIONID), date (‘date’, we assumed local time at sample site and reported in UTC) date and time (‘date_time’, we assumed local time at sample site and reported in UTC), value of the result (FINAL for ‘result_va’, concentration of pigment), units of the result (UNITS for ‘result_units’, as µg/L or mg/m^2^). Note that there were no fields for ‘remark_cd’, ‘sample_depth’, ‘hydrologic_event’ in the NWQL data.

Finally, in the WQP data, it is not clear if corrected chlorophyll *a* values are from the standard EPA 445 method, or the modified method. Multiple WQP records reported “Corrected chlorophyll *a* by EPA 445”, though this phrase has ambiguous meaning. It could mean “chlorophyll *a* corrected for pheophytin” or “chlorophyll *a* based on narrow band fluorometry”. It was not possible to differentiate between these two methods from the WQP metadata. Previous publications have emphasized that measurement and reporting of “corrected chlorophyll *a”* (EPA 445) be discontinued, in favour of “uncorrected chlorophyll *a*”^34,37^ or “chlorophyll *a* based on narrow band fluorometry”. Future work could resolve the discrepancy in reporting.

### Verification

Some site records had the same date and had frequent times (for example, every hour, or every 15 minutes) and reported a method code that was inconsistent (EPA 445) with a discrete sample. We interpreted these records as being from *in situ* sensors and deleted them, where we could find them. It is possible that *in situ* sensor data appear elsewhere in the WQP as records with an incorrect ‘method_cd’.

Methods (from ‘method_cd’ column) after harmonization still contained many codes that were accepted as being measures of chlorophyll *a* concentration by spectrophotometry or fluorometry, including values of “NA”. In a few cases, a code for hydrologic event was included by original data provider, which provides a link between pigment concentration and storms, algal blooms, tidal action, ice cover, and other events.

### Duplicate record removal

Data from the two sources were combined into a single dataset by a simple merge of the rows, based on common column content. We checked for duplicate records and removed those values by exact matches across these five columns, ‘site_no’, ‘date’, ‘date_time’, ‘parameter’, and ‘censored_cd’ (indicating values were below a detection limit). If there was a duplicate, the WQP record was retained over the NWQL record because it contained sample depth information. A special exception was made for two duplicate records because the NWQL data had a ‘censored_cd’ of ‘ < ‘ but the equivalent WQP record did not. In these two instances, the NWQL records were kept instead of their WQP counterparts. NWQL records for sites that were not recognizable NWIS sites were removed because their location could not be verified.

### Site metadata

Site information for unique sites in the final harmonized, filtered, and combined dataset was downloaded using the USGS R package dataRetrieval (v2.7.12). Fields were renamed into nine columns described in the entity-attribute information^1^. Some of the original WQP values from the ‘site_type’ column were combined to reduce the number of unique possible site types from 31 to 21. More information about how these were combined is available in the entity-attribute section for the site metadata file^1^.

## Data Records

Data are stored in the USGS ScienceBase repository^1^ and are composed of five files. The first file, ‘national chlorophyll data metadata.xml’, is a detailed compilation of identification, data quality, entity and attribute, distribution and metadata reference of the source data. The second file, ‘national chlorophyll site metadata.csv’ is described (Table 4). Three files contain the data (corrected chlorophyll a, pheophytin, uncorrected chlorophyll a) and are structured in the same manner (Table 5).

**Table 4:** The ‘national chlorophyll site metadata.csv’ file includes 12 fields, as described below.

Field	Field Description	Example Entry
org_cd	Organization code	NARS_WQX
site_no	Site number	SOUTHUTE-AR 15-4
site_name	Site name	Turquoise Lake
site_type	Site type	River/Stream
huc08	Hydrologic unit code 8*	14030002
county	County	Palm Beach County
state	State	Louisana
latitude	Latitude (decimal degrees)	18.45611
longitude	Longitude (decimal degrees)	−66.1083
horizontal_datum	Horizontal datum**	NAD83
elevation	Elevation (m)	26.811
vertical_datum	Vertical datum***	NGVD29

*Hydrologic unit codes are delineated and georeferenced to U.S. Geological Survey 1:24,000 scale base maps. HUC 8 represents the subbasin level, roughly medium size river basins.

**The North American Datum of 1983 is the horizontal control datum for the U.S.

***The vertical datum is the surface of zero elevation to which heights of sample collection are referenced. The National Geodetic Vertical Datum of 1929 (NGVD29) and the North American Vertical Datum of 1988 (NAVD88) are most common, but others are used.

**Table 5:** Three data files, ‘national chlorophyll corrected chlorophyll a data.csv’, ‘national chlorophyll pheophytin data.csv’ and ‘national chlorophyll uncorrected chlorophyll a data.csv’ include the same 14 fields, as described here.

Field	Field Description	Example Entry
source	Data source	NWQL
site_no	Site number	SOUTHUTE-AR 15-4
date	Date	dd/mm/yyyy
date_time	Date and time (UTZ)	yyyy-mm-ddT00:00:00Z
parameter	WQP CharacteristicName	Chlorophyll *a* - Periphyton (attached)
pigment	Pigment type	Corrected chlorophyll a
result_va	Result value	6.29
result_units	Result units	µg/L
sample_depth	Sample depth (m)	1
censored_cd	Censored code*	Not censored
remark_cd	Remark code**	Final
method_cd	Method code***	445.0 ~ EPA; Chlorophyll and Pheophytin in Algae by Fluorescence
result_qualifier_cd	Optional, open comment	Dark, tannic water
hydro_event_cd	Optional, fixed entry	Flood

*Censored codes are described in the section on Harmonization.

**Remark code in Indicates the acceptability of result, with respect to QA/QC.

***Method used for analysis, specific to the data contributor.

**Fig. 2:**
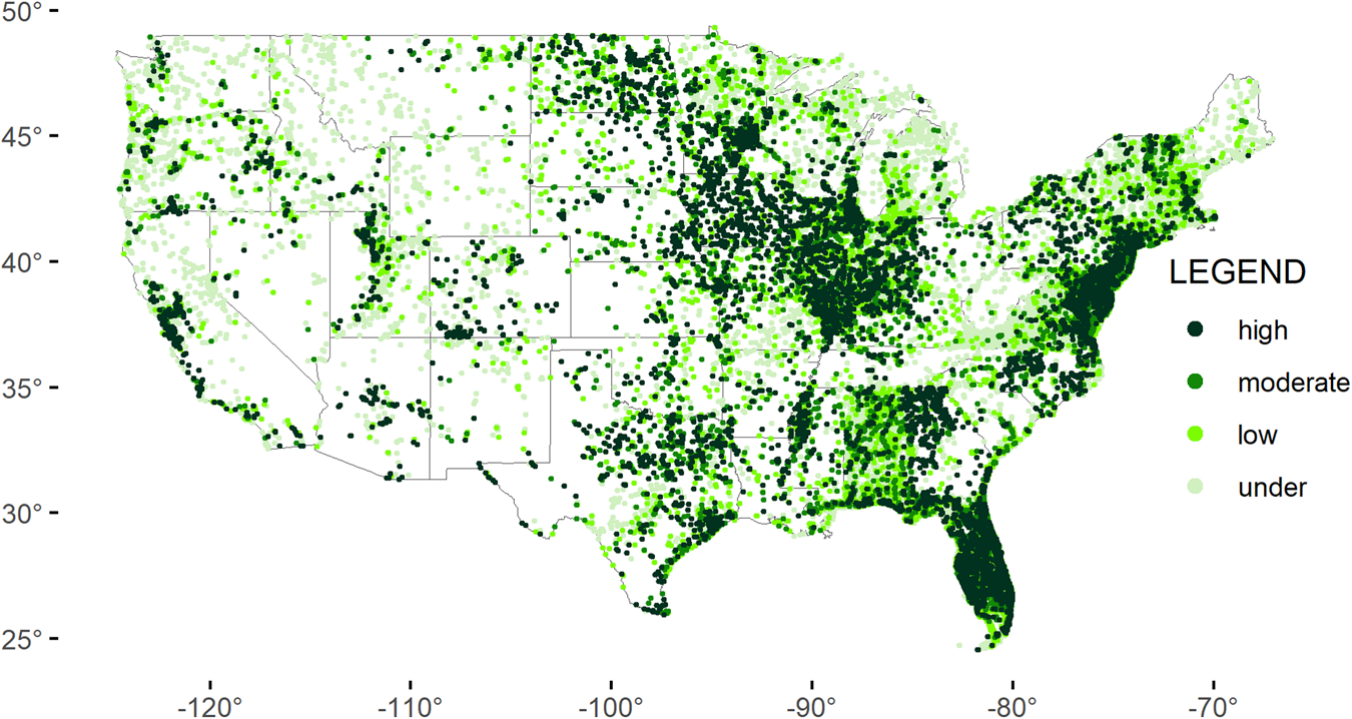
Map of the continental United States showing records for corrected chlorophyll *a* and uncorrected chlorophyll *a* from phytoplankton (µg/L), based on threshold values: under (<10 µg/L), low (10–25 µg/L), moderate (25–50 µg/L), and high (>50 µg/L).

**Fig. 3:**
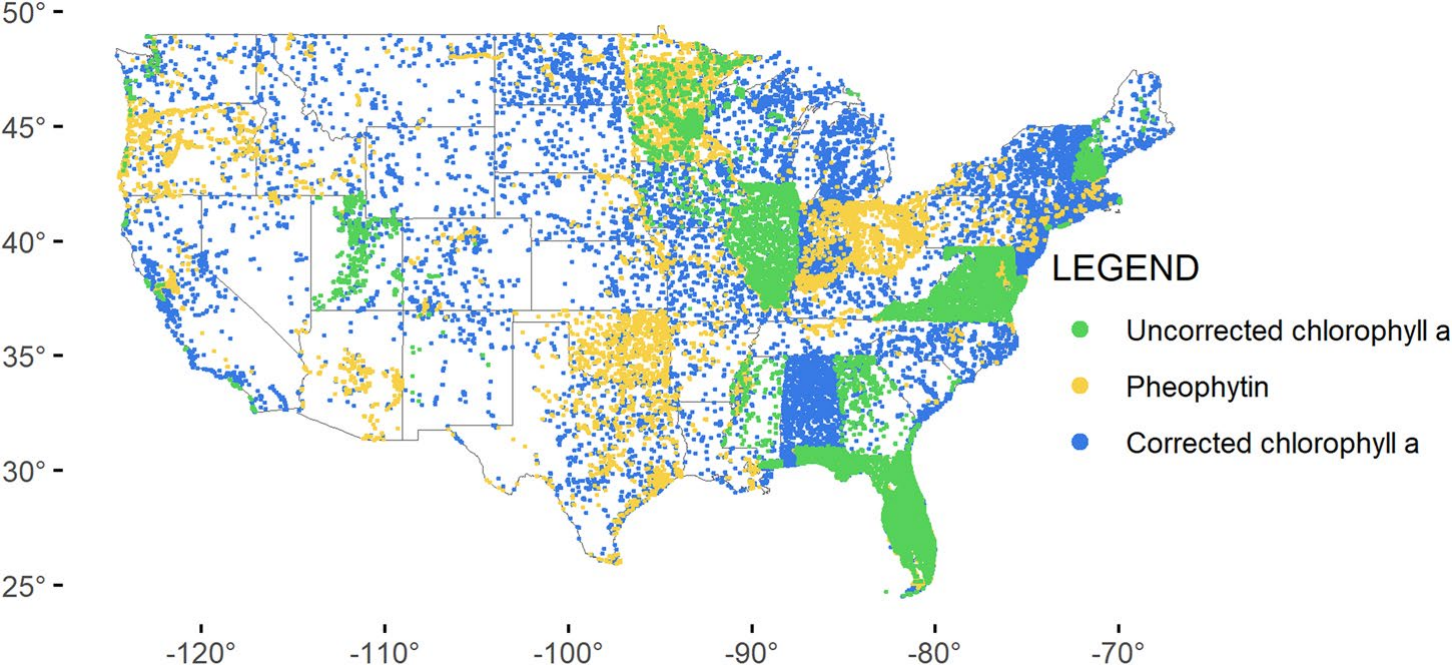
Map of the continental United States showing phytoplankton records, color coded by pigment as uncorrected chlorophyll *a* (green), pheophytin (yellow), and corrected chlorophyll *a* (blue).

**Fig. 4:**
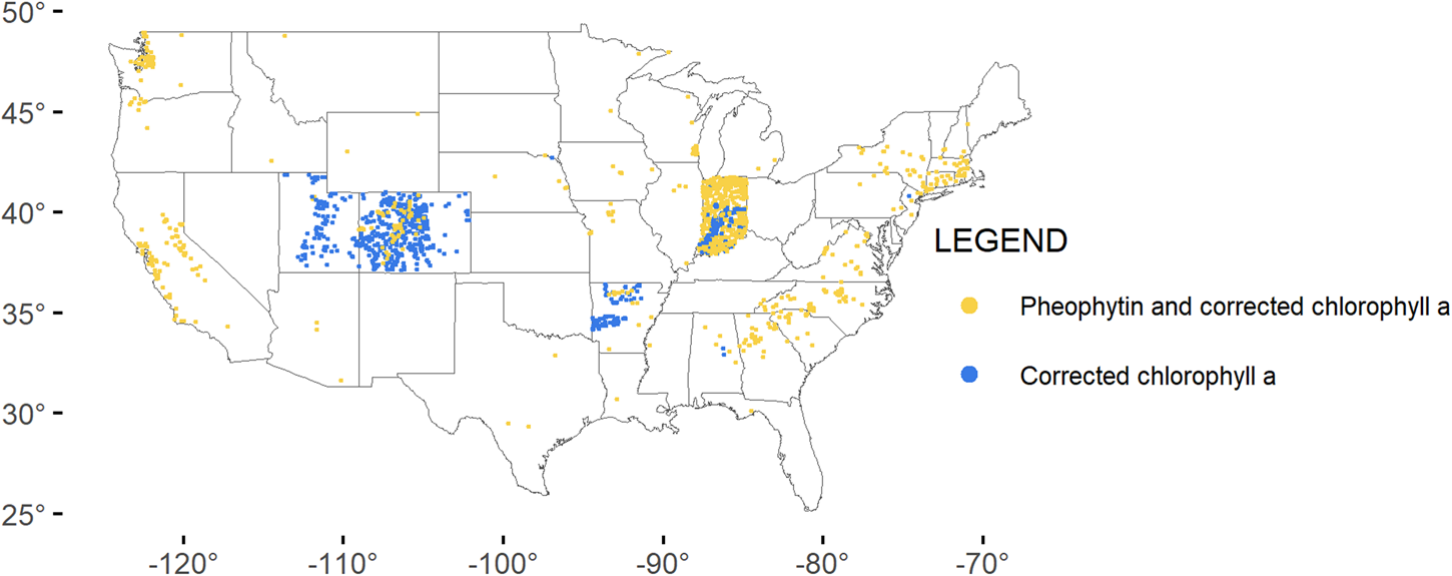
Map of the continental United States showing periphyton records, by pigment as pheophytin and corrected chlorophyll a (yellow) and corrected chlorophyll *a* (blue).

**Fig. 5:**
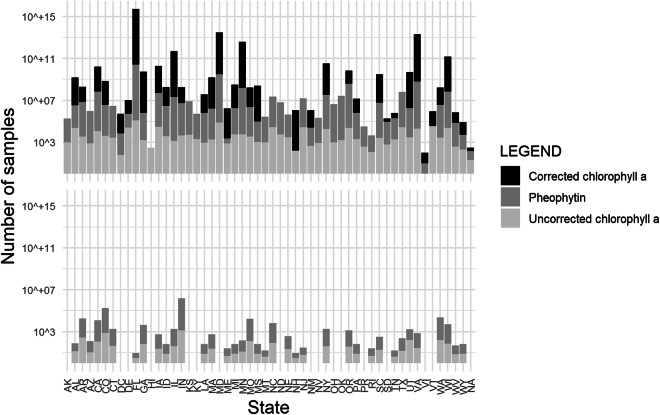
Plots showing the state (abbreviation) on the x-axis, by the number of samples on the y-axis (log base 10) for each pigment type: uncorrected chlorophyll *a* (light gray), pheophytin (gray), corrected chlorophyll *a* (black). The last value label on the x-axis “NA” is for samples that lacked a reported state. The top plot shows the number of phytoplankton samples and the bottom plot shows the number of periphyton samples.

A map of the continental US shows the concentrations of uncorrected and corrected chlorophyll *a* in phytoplankton samples (Fig. 2). High concentrations (50 µg/L and above) occur across midwestern states, southeast states and coastal areas, northeast coasts, and Florida. States vary in collection frequency, phytoplankton vs. periphyton sampling, distribution of sample collection, and reporting, as evidenced by the geographic distribution of pigment types for phytoplankton and periphyton (Figs. 3,4). Furthermore, sample density across states is not evenly distributed, with great variation in the number of samples collected and pigment types reported (Fig. 5). Florida has the greatest number of phytoplankton samples within the dataset, followed by Virginia and Maryland. Indiana has the greatest number of periphyton samples within the data, followed by Colorado.

**Fig. 6:**
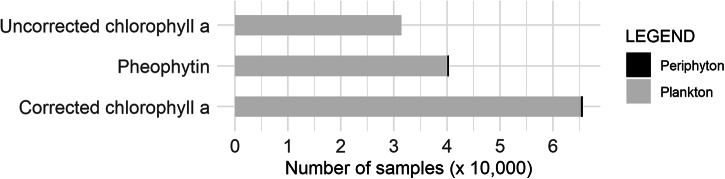
Number of samples (× 10,000) for each pigment type indicating phytoplankton (gray) and periphyton (black).

**Fig. 7:**
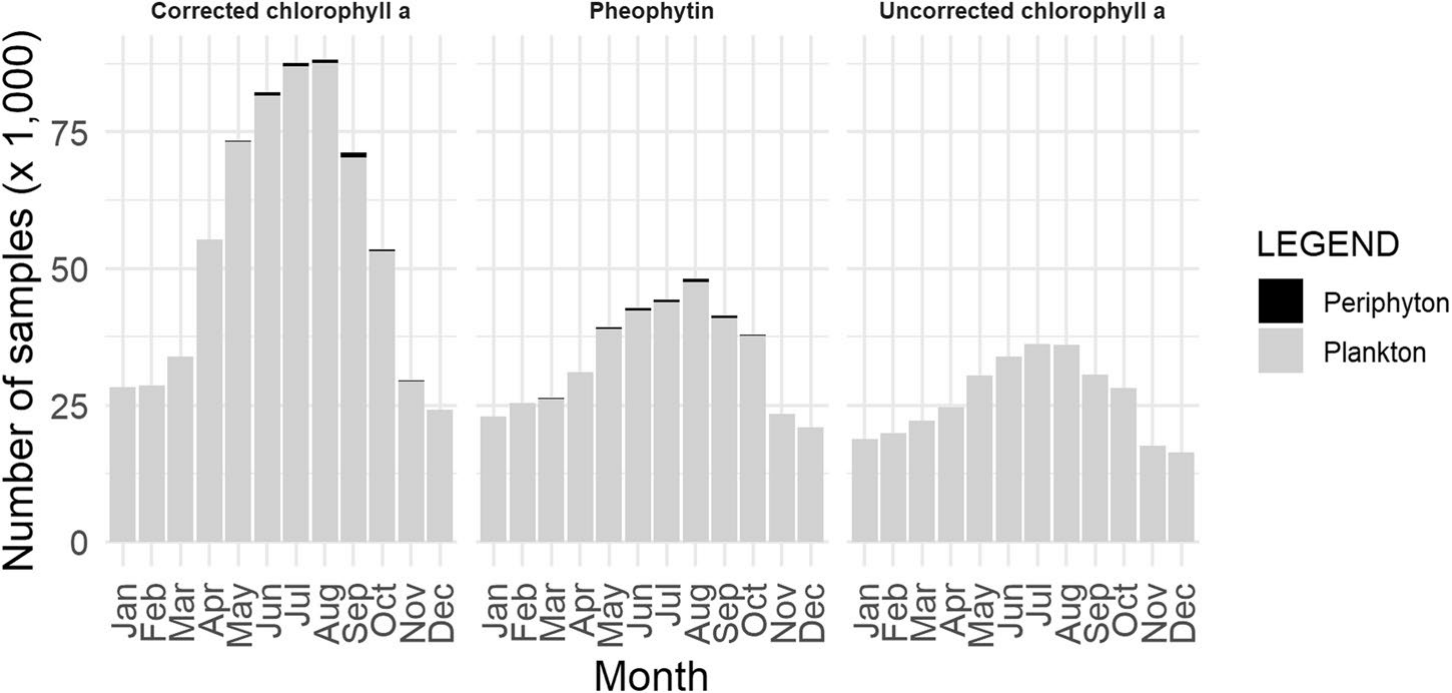
Number of samples (× 1,000) by month for phytoplankton (grey) and periphyton (black).

**Fig. 8:**
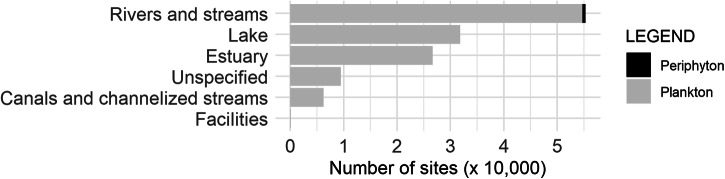
Number of sites (× 10,000) from rivers and streams, lakes, estuaries, unspecified water bodies, canals and channelized streams, and water treatment facilities with phytoplankton (grey) and periphyton (black) samples.

The greatest number of records are for samples from the phytoplankton for corrected chlorophyll *a* (651,242), followed by pheophytin (406,056), and finally, uncorrected chlorophyll *a* (315,492) (Fig. 6). A small fraction of records is from the periphyton, with corrected chlorophyll *a* (2,236), pheophytin (1,187), and no values were reported for uncorrected chlorophyll *a*. While the number of records is greatest for samples collected during warmer months, there are records throughout the year (Fig. 7). Most of the samples in the dataset were collected in rivers and streams, followed by lakes, then estuaries, and finally other water bodies including unspecified water, canals and channelized streams, and water treatment facilities (Fig. 8). While phytoplankton samples were from all habitat types, periphyton samples were restricted to rivers and streams.

After removing the records from sites that were suspected to be from *in situ* sensors (inappropriately submitted to WQP as discrete chlorophyll samples), the dataset includes 83,829 unique site names. However, in this dataset, co-located sites were not aggregated, for example, multiple site numbers from an individual lake, a specific river reach, or geographic location were not combined. Note that site numbers were created by the collecting organization, some of which do not use the same site number over multiple years or for different sampling campaigns, even if the location is the same. Aggregation of samples would likely increase the density of samples at physical location.

**Fig. 9:**
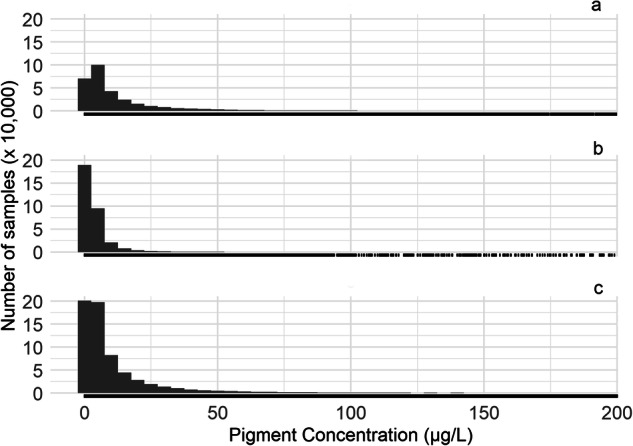
Number of phytoplankton samples (× 10,000) with pigment concentration (µg/L) of (**a**) uncorrected chlorophyll *a*, (**b**) pheophytin, and (**c**) corrected chlorophyll *a* (samples with concentrations below 200 µg/L). The bin width is equal to 5. The rug plot (below) indicates the occurrence of at least one sample at that concentration.

**Fig. 10:**
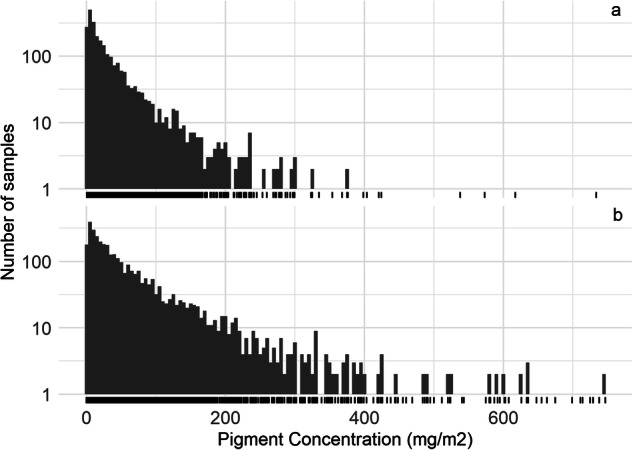
Number of periphyton samples (y-axis log base 10) with pigment concentration of (**a**) pheophytin, and (**b**) corrected chlorophyll *a* (samples with concentrations below 750 mg/m^2^). The bin width is equal to 5. The rug plot indicates one sample at that concentration. Concentrations of periphyton can reach very high levels, but a high benthic concentration of pigments does not necessarily reflect high growth rates, because biomass accumulates over time.

The concentration phytoplankton pigments (by volume) and periphyton pigments (by surface area) had extreme ranges. Concentration of planktonic pigments are shown for values below 200 µg/L (Fig. 9). While most phytoplankton samples have low concentrations (below 10 µg/L) of chlorophyll pigments, 6,000 records were extreme, at greater than 2000 µg/L (0.48% of all records). We verified that some high sample concentrations were from algal bloom surface “scums”. Concentrations of periphyton chlorophyll can reach much higher values than the planktonic chlorophyll (Fig. 10), because attached algae accumulate biomass in the absence of scouring flows, for example under drought conditions. Accordingly, periphyton pigment concentrations can also reach high values, but in absence of a “bloom”. Periphyton concentrations are shown for values up to 750 mg/m^2^ (Fig. 10).

## Technical Validation

Values within WQP fields were checked for correct values. Invalid or inappropriate entries were removed. Records with pigment concentrations that were not integers or had associated censored codes were removed. Records with negative pigment values were removed. *In situ* probes and nonsensical methods were identified by evaluating the related method code; records with any such method were removed. Records from the organization “CSKTRIBE” were removed, because of a known conversion error. A site in British Columbia, Canada, was removed since it fell outside the United States and its territories.

Values within NWQL fields were checked for correct values. Invalid or inappropriate entries were removed. Data were filtered and replicates (‘SAMP TYPE’ = 7) were removed. Records that were not one of 14 desirable ‘MEDIUM’ codes were removed. Records with medium codes that were retained included ‘WS’ (surface water), ‘WSQ’ (surface water quality control),‘BP’ (plant tissue), ‘BPQ’ (plant tissue quality control), ‘BH’ (phytoplankton), ‘BHQ’ (phytoplankton quality control), ‘BY’ (phytoplankton), ‘BYQ’ (phytoplankton quality control), ‘BE’ (periphyton), ‘BEQ’ (periphyton quality control), ‘BD’ (periphyton),‘BDQ’ (periphyton quality control), ‘SB’ (bottom material), and ‘SBQ’ (bottom material quality control).

## Usage Notes

Extreme values for uncorrected and corrected chlorophyll *a* remain in the dataset. Users should be aware of these extremely high concentrations reported from some sites and regions. Initially, we planned to omit values over a certain threshold from the data as being erroneous. But because we were able to verify many records as being from surface algal scums, we suggest that users confirm extreme values with the data provider. As mentioned earlier, we removed “CSKTRIBE” samples due to known errors after communicating with the data originators, but we were not able to contact all data providers to verify high pigment concentrations. A small number of algal blooms (437) were marked by contributors and can be confirmed by ‘hydro_event_cd’ containing “algal bloom”.

Two organizations, (with ‘org_cd’ of “MDE_FIELDSERVICES_WQ” and “21FLHILL_WQX”), reported, in some cases, a Characteristic Name of ‘Chlorophyll’, that represented a sum of values from different pigment types (Table 1).

**Fig. 11:**
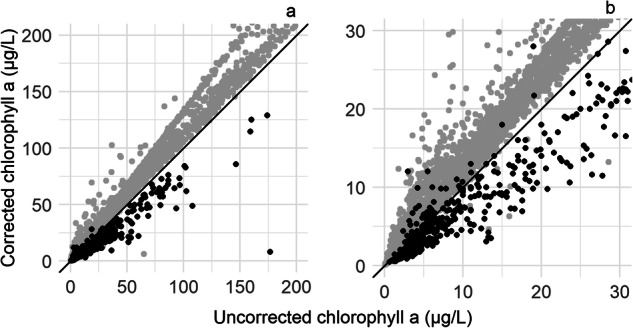
Relation between uncorrected chlorophyll *a* and corrected chlorophyll *a* for samples that had results for both pigment types at the same site number, day, and time. Values (**a**) from 0 to 200 µg/L and (**b**) from 0 to 30 µg/L. Points in gray represent values from organizations that used the Characteristic Name ‘Chlorophyll’ to include a sum of pigment types (see Table 1). In general, values of corrected chlorophyll *a* that are greater than their corresponding uncorrected chlorophyll *a* value indicate the presence of interferences from other pigments. In the absence of interferences, corrected chlorophyll *a* values fall below the uncorrected chlorophyll *a* values (i.e., the 1:1 line).

Records from the same day and time, with all three pigment types, reinforce the argument that uncorrected chlorophyll *a* values be used over corrected chlorophyll *a*, because the corrected value is subject to interferences^15,37^ (Fig. 11). Samples with corrected chlorophyll *a* values greater than the corresponding uncorrected chlorophyll *a* value, indicate the presence of interferences from other pigments. In the absence of interferences, corrected chlorophyll *a* values would fall below the uncorrected chlorophyll *a* values (i.e., the 1:1 line).

Temporal series of records from sites with both uncorrected chlorophyll *a* and pheophytin offer the opportunity to determine periods of growth and senescence of algae in planktonic habitats. These records could be informative in determining the timing of peak growth in such sites.

This manuscript reflects the dataset reviewed and released in 2023^1,39^. The dataset may be periodically updated with new data, generating sequential versions of the ScienceBase data release.

## Data Availability

There are no restrictions to use of the data, which are publicly available (10.5066/P9J0ZIOF)^1^ as well as the code to produce the data (10.5281/zenodo.7879199)^39^.
